# Allocating health care resources in jails and prisons during COVID-19: a qualitative study of carceral decision-makers

**DOI:** 10.1093/haschl/qxae015

**Published:** 2024-02-14

**Authors:** Brandon Doan, Camille Kramer, Brendan Saloner, Minna Song, Carolyn B Sufrin, Leonard S Rubenstein, Gabriel B Eber

**Affiliations:** Department of Health, Behavior and Society, Johns Hopkins School of Public Health, Baltimore, MD 21205, United States; Department of Gynecology and Obstetrics, Johns Hopkins School of Medicine, Baltimore, MD 21205, United States; Department of Health Policy and Management, Johns Hopkins Bloomberg School of Public Health, Baltimore, MD 21205, United States; Department of Health Policy and Management, Johns Hopkins Bloomberg School of Public Health, Baltimore, MD 21205, United States; Department of Health, Behavior and Society, Johns Hopkins School of Public Health, Baltimore, MD 21205, United States; Department of Gynecology and Obstetrics, Johns Hopkins School of Medicine, Baltimore, MD 21205, United States; Department of Epidemiology, Johns Hopkins Bloomberg School of Public Health, Baltimore, MD 21205, United States; Department of Epidemiology, Johns Hopkins Bloomberg School of Public Health, Baltimore, MD 21205, United States

**Keywords:** jails, prisons, incarceration, resource allocation, health care, COVID-19, pandemic

## Abstract

COVID-19 created acute demands on health resources in jails and prisons, burdening health care providers and straining capacity. However, little is known about how carceral decision-makers balanced the allocation of scarce resources to optimize access to and quality of care for incarcerated individuals. This study analyzes a national sample of semi-structured interviews with health care and custody officials (*n* = 32) with decision-making authority in 1 or more carceral facilities during the COVID-19 pandemic. Interviews took place between May and October 2021. We coded transcripts using a directed content analysis approach and analyzed data for emergent themes. Participants reported that facilities distributed personal protective equipment to staff before incarcerated populations due to staff's unique role as potential vectors of COVID-19. The use of testing reflected not only an initial imperative to preserve limited supplies but also more complex decision-making about the value of test results to facility operations. Participants also emphasized the difficulties caused by limited physical space, insufficient staff, and stress from modifying job roles. The rapid onset of COVID-19 confronted decision-makers with unprecedented resource allocation decisions, often with life-or-death consequences. Planning for future resource allocation decisions now may promote more equitable decisions when confronted with a future pandemic event.

## Introduction

The COVID-19 pandemic highlighted key resource deficiencies in carceral health care. These deficiencies, including constraints on medical supplies and appropriate staffing levels, likely contributed to the significant health disparities experienced by incarcerated people.^1-3^ During the height of the pandemic, people in prison were 5.5 times more likely to be infected with COVID-19 and 3 times more likely to die from COVID-19.^3^ Despite being exceptionally dangerous, carceral facilities were not prioritized like nursing homes or other high-risk congregate living spaces.^4,5^ Similarly, when vaccines became available, incarcerated people were generally prioritized separately (and often later) than age-matched nonincarcerated persons.^6^ The lack of resources contributed to beliefs among some incarcerated patients that they were being punished and denied adequate resources.^7^

Access to and quality of health care in carceral facilities depend upon maintaining inventories of necessary supplies and equipment. Medical operations also depend upon leadership structures that empower decision-makers during times of crisis. This leadership, often in combination with external oversight and accreditation to systematically monitor the quality of care within jails, is key to implementing novel solutions to the challenges posed by the pandemic.

This qualitative study explores how carceral decision-makers allocated key health resources during the COVID-19 pandemic, including medical supplies, such as masks and vaccines, and more intangible resources such as staff morale. We define resource allocation as the decision-making processes through which leaders determine how scarce health-related resources are made available, and how those decisions are implemented.^8^ Understanding how carceral facilities allocate resources during a public health emergency is essential to advance equitable health care for people who are incarcerated and to aid in planning for the next disaster or pandemic.

## Data and methods

We conducted semi-structured, qualitative interviews with a national convenience sample of officials from May to October 2021. Participants were recruited through emails to members of the National Commission on Correctional Health Care listserv, the American College of Correctional Physicians, and other health and custody officials known to study team members. For inclusion in the study, individuals were selected for their decision-making authority at 1 or more carceral facilities during the COVID-19 pandemic. Geographic location was not a barrier, given that we conducted interviews virtually. Participants were not offered compensation for their participation.

Interviews were conducted via video-conferencing and simultaneously audio-recorded, except for 1 interview in which only written notes were taken. One team member (C.K.) led every interview with other team members co-interviewing (B.S., G.B.E., C.B.S., L.S.R., M.S.). Interviewer expertise ranged from public health, health policy, law, anthropology, bioethics, and carceral health care backgrounds. All interviewers were trained in qualitative research methods. Interviews lasted approximately 60 minutes. We developed a semi-structured interview guide asking respondents to recount the chronology of how their facility or organization became aware of COVID-19, initiated a response plan related to quarantine and isolation, dealt with cases when they emerged, and planned for the easing of containment measures. We asked questions to probe both the substantive outcomes of decision-making and the decision-making process, including the sense that equitable decisions could be reached within the facility's capacity vs the sense that it was not possible to achieve fair outcomes (ie, moral distress) leading to unavoidable ethical dilemmas.^9^ We also asked a series of overarching questions to elicit reflections on the decisions they were forced to make and on the broader successes and failures of COVID-19 containment. We stopped recruitment once thematic saturation was met based on similar responses from multiple participants.

Interview recordings were professionally transcribed and uploaded into Dedoose software (manufactured by SocioCultural Research Consultants, LLC) except for 1 interview captured by typed notes.^10^ We coded transcripts using a directed content analysis approach, revising our initial a priori, inductive codebook in an iterative process after gathering data from participants.^11^ Two team members (M.S., B.D.) met to review transcripts and discuss differences in coding until they reached agreement. We then analyzed data for emergent themes related to resource allocation and COVID-19 treatment and prevention. We followed the Standards for Reporting Qualitative Research when reporting findings.^12^ The study protocol was approved by the Johns Hopkins Bloomberg School of Public Health Institutional Review Board.

## Results

We conducted 26 interviews with 32 individuals who came from a range of geographic regions and types of carceral facilities. Twenty-one interviews were 1 on 1 and 5 were group interviews with leadership teams working at the same facility. Twenty-five interviewees were health care administrators or clinical leads and 7 were high-ranking custody officials. Most participants had decision-making authority within a facility or in a department. Several operated at the state level or across multiple states. The majority of participants worked in jails as opposed to other carceral facilities, were involved in health care operations, and were located in the western United States (Appendix A). Key themes emerged around 6 resources: (1) hospitalization, (2) masking, (3) physical space, (4) vaccination, (5) testing, and (6) staffing. A complete table of quotes corresponding to each resource is provided in Appendix B.

### Sending patients out for hospitalization during the pandemic required balancing the welfare of individual patients with broader considerations about staffing and risk of spreading COVID-19

Some health care decision-makers expressed concerns about moving patients from jails and prisons into community hospitals due to the risk of spreading COVID-19 into the community. As 1 participant observed, “We try to keep as many people within the facility so that we’re not spreading things.” Only patients with serious medical emergencies such as needing 24/7 oxygen support were considered for hospitalization at that facility. Relatedly, decision-makers weighed the benefits of hospitalization vs the risk of patients contracting COVID-19 outside, and potentially bringing it back to their facilities: “Unless it was essential or an emergency, we didn’t want to transport somebody out and them get exposed to COVID and bring it back into the jails.”

These decisions were made against the backdrop of societal norms that tend to devalue the health of incarcerated people, including what 1 official perceived to be “a community bias to save beds for non-incarcerated individuals.” Participants reported that patient transport to and from community hospitals, which did not accept them or returned them after insufficient care (eg, with still-low oxygen levels), led to delays in proper care, including death for some incarcerated patients (Appendix B, Quote 1). From a practical perspective, decision-makers also considered the extra time it would take to negotiate with hospitals to accept their patients vs trying to treat patients themselves (Appendix B, Quote 2). In terms of process, respondents often described themselves as being in the position of advocating externally for their patients, but ultimately needing to accept the decisions made by outside health providers.

Across facilities, officials also considered staffing shortages that impeded sending patients out for hospitalization. Facilities generally require custody officers to accompany patients when they leave the facility for off-site care. Sending custody officers out to escort patients removes them “away from stuff that they could do at the prisons,” many of which were already significantly understaffed. The logistical considerations and the high-stakes implications of the decisions made allocation of hospitalization one of the most commonly cited resource challenges among respondents.

### Decision-makers distributed limited supplies of masks to staff before incarcerated populations

During the first year of the COVID-19 pandemic, perceived higher-quality masks (eg, N95 vs cloth masks) were difficult to obtain. With limited supply, decision-makers prioritized N95 masks to staff interacting with COVID-19–positive patients and staff interfacing with the community (eg, officers escorting patients to community hospitals). As 1 respondent stated, they did so “for the benefit of hospital staff, as well as [their] own” to avoid outbreaks. In some facilities, this caused some internal tension, including custody officers who threatened to go on strike if they did not also receive N95 masks (Appendix B, Quote 3); non–COVID-19 patient interfacing officers and remaining staff were provided with lesser, but still effective, KN95 masks when available. The process leading to these decisions was sometimes described in terms of accommodation to security rather than explicit weighing of public health imperatives.

From experience, many decision-makers communicated the risks of wearing unfitted N95 masks, citing their level of protection as “no better than surgical masks.” However, with limited supply, decision-makers worked with “whatever N95s we could get.” Many times this meant that staff wore any N95 masks vs masks that fit them in compliance with Occupational Safety and Health Administration regulations. One participant even described custody officers re-wearing masks for weeks due to mask shortages as their biggest physical resource allocation challenge: “Officers would use the same mask for weeks because we couldn’t get any. That was my biggest, roughest thing.” To increase compliance with mask mandates, which many facilities struggled to enforce due to the politicization of the pandemic, other decision-makers allowed their staff to wear whatever masks they wanted (Appendix B, Quote 4).^13^

When mask inventory increased, many facilities disseminated masks (notably, those perceived as less effective) to incarcerated populations. They also implemented new masking guidelines to varying degrees of success: “After about a month or two, we started requiring masks of the inmates as well. And that worked so-so. They were cloth masks. They didn’t fit that well. And people struggled a little bit with that.” Despite some facilities enforcing mask mandates on incarcerated populations at all times, decision-makers understood that “wearing a mask all the time is just not a reality in a correctional facility.”

### Participants viewed physical space, its openness and ability to be transformed into makeshift treatment zones (eg, for quarantine or isolation), as an invaluable resource for pandemic control in centers of detention

One participant likened rationing physical space for quarantined or isolated patients to “a giant game of chess.” With limited space, participants had to consider “custody overrides, where you’re mixing folks from different custody levels based on their COVID status rather than their custody status.” This “game of chess” required meeting challenges such as gang-affiliated and other violent behavior (Appendix B, Quote 5). Relatedly, some participants weighed the benefits of aggressively isolating patients vs the complication of transfers, which risked spreading infection. These highly impactful decisions were made internally to carceral leadership, however, and participants did not mention seeking perspective or advice from incarcerated people.

Some facilities expanded upon pre-existing health emergency plans for isolating people (eg, during a bad flu season or during the H1N1 outbreaks).^14^ However, COVID-19 required additional transformation and repurposing of physical spaces: “We did have a pandemic plan in place for having to isolate people. You know, like if we had a particularly bad flu season. We have a wing of a living unit at [facility] that’s meant to accommodate 30 sick patients at a time. We had hundreds. So we actually used our gymnasium as sort of a temporary isolation location. We also used the visitation room. I mean our staff got very creative, for sure.”

Perhaps the most transformative system-wide adjustments intended to open physical space were decarceration policies that reduced population overcrowding and allowed for greater social distancing in facilities.^15^ While these policies were generally created outside of the facility (eg, by state legislatures) and there was often limited input provided by carceral decision-makers into the decarceration process, complex implementation challenges fell to the facility leadership. In some cases, leaders were criticized for rushing incarcerated individuals out of the system too quickly. For example, 1 respondent expressed concern that rapidly releasing patients with opioid use disorder could lead to heightened overdose risk if the patients were not continued on medications provided during incarceration (Appendix B, Quote 6).

One noted advantage of the sweeping decarceration policies was decreasing the demand overload for internal medical services: “The good news was this big decarceration that happened. There was a massive efflux, and with that came much reduced demand for access. You know, there just were less sick calls being made because there were less people here. So it actually kind of worked out and was actually kind of manageable.”

### When COVID-19 vaccines became available, administrators often prioritized vaccines to staff over incarcerated persons, but these allocation decisions were relaxed over time

Vaccines to prevent serious COVID-19 illness were initially authorized and produced in limited quantities in early 2021. Health care decision-makers were faced with the decision on how to allocate doses to staff, incarcerated persons, or some combination of the 2.^6^ While the exact criteria and phasing varied across facilities, respondents generally described vaccine rollout going to staff first given their unique role as potential vectors of the virus, moving in and out of facilities. One official explained their highest vaccine priority going to health care workers due to their closest proximity to sick patients (Appendix B, Quote 7). The second highest vaccine priority at this facility went to custody officers working in clinical spaces. The third vaccine priority among staff generally was allocated to custody officers who worked in other “first responder” roles in the community (eg, police officers, fire marshals, etc). The remaining staff and incarcerated persons were the last groups to receive the vaccine.

Despite states placing incarcerated populations at a lesser vaccine priority, most decision-makers felt that they had a responsibility to provide resources to protect these people against a virus that they were exposed to through no fault of their own (Appendix B, Quote 8). One participant articulated that “when you have a pandemic type situation, separating a community from a prison doesn't really work because most people that go to prison are going to get out. And really, the vast majority of them get out relatively soon. If you don't take care of people in prison, it will affect the community as well.” Meanwhile, decision-makers also acknowledged “there being a moral and ethical thing to take care of the inmate” when deciding to ration vaccines for people who are incarcerated, against community pressure to reserve vaccines for people who are not incarcerated.

While limited supply was generally a constraint on broader provision of vaccines, 1 official reported that they focused first on prioritizing people with pre-existing serious chronic illnesses, despite having sufficient supply to vaccinate more widely. They did this so as not to face outside community scrutiny for “giving advantages to people who are incarcerated” (Appendix B, Quote 9). In other words, they had enough vaccines to vaccinate additional incarcerated individuals who, if not incarcerated, would qualify, but they did not want to make it seem like incarcerated individuals were better off than non-incarcerated individuals.

### The use of COVID-19 testing reflected not only an initial imperative to preserve limited supplies but also more complex decision-making about the value of test results to facility operations

When COVID-19 rapid tests became available, many health care leaders first distributed their limited supply to symptomatic patients over asymptomatic patients: “In the beginning, we didn’t test a lot of people. We only tested the very symptomatic ones. That changed over time.” As pathways for COVID-19 transmission became better understood, many decision-makers began implementing mass testing of entire facility cohorts. For example, in 1 facility, “if there were two people that were positive for COVID in the facility—then, the whole facility would be tested.” According to an official at this facility, “once we started doing that, we found lots of people who were asymptomatic who were positive.”

Some decision-makers implemented mandatory testing for all incoming transferred patients. They did so to isolate patients sick with COVID-19 appropriately and minimize the risk of viral transmission in their facilities. This testing decision had broader resource allocation consequences, including impacts on staff charged with performing the tests who were removed from their regular duties. In making this decision, they also weighed resource constraints on local law enforcement, who had to stay longer in booking and took longer to return to street patrol (Appendix B, Quote 10).

Finally, there was a perception among some decision-makers that the choice not to test (in other facilities) stemmed from a desire not to draw attention to the scope of outbreaks. One decision-maker conveyed, “Of course, if you don't test, then you don't have a problem” and attributed non-testing policies either to facilities being dishonest or not paying enough attention: “Some facilities were honest and other facilities just weren’t. I found that that was the biggest issue, was some facilities just didn’t pay attention. I don’t know if they didn’t care but they didn’t pay attention and thought they didn’t have a problem.” Implicit in this comment is the idea that the process of testing in many facilities was guided as much by optics as by public health considerations (although no respondent admitted this applied in their own circumstances).

### Participants emphasized the numerous difficulties caused by insufficient staff subject to stress from understaffing and modified job roles

In their COVID-19 control strategies, many decision-makers placed unprecedented responsibilities on custody officers, contributing to widespread burnout and moral distress.^9^ As noted, the workload was heavy and included converting gyms and libraries into nontraditional housing units or creating new patrol stations. Tensions also rose between custody and health care staff as their roles conflicted (Appendix B, Quote 11). These changes in roles were compounded by staff absences due to illness, family obligations, and other outside duties that left facilities severely understaffed.^8^ As 1 participant emphasized, “The number one concern right now is staff burnout in combination with the vaccine mandate creating even further short staffing than we had to begin with.” To maintain staffing levels as high as possible while navigating the politicization of the pandemic, other facilities opted not to enforce vaccine mandates.^16^

Many facilities were also faced with prioritizing care for patients with COVID-19 over non-pandemic, routine health services such as sick call and care for dental and chronic conditions. In part, this was due to staffing shortages. In effect, care for patients with COVID-19 created a secondary health care crisis with a significant backlog for those needing more routine care (Appendix B, Quote 12). Participants simultaneously reported stress from understaffing, which perpetuated basic concerns about keeping operations going: “As soon as people start getting COVID, how are you going to serve the community? How are we going to serve our jail? How are we going to keep these operations going? We couldn’t take time off. We couldn’t work from home. We had to be here for the community. We had to be here for the jail. And so, that was what kept me up at night is wondering if I was going to have the staff the next day to run operations.”

## Discussion

COVID-19 created profound resource allocation challenges in carceral facilities. While some of these challenges directly pertained to the allocation of tangible resources such as personal protective equipment (PPE) and hospital beds, many of the challenges were related to intangible resources such as staff morale. In deciding who received what resources and when, participants sometimes justified their decisions as actions that would minimize risk, although they often balanced risk mitigation with other practical and political considerations.

These decisions, however, typically fell short of applying ethical standards for prioritization that existed during this time in community settings. While meeting the community standard for incarcerated people (often referred to as “equivalence”) is both a human rights norm outside of the United States and often a basis for legal entitlement, our US-based respondents did not invoke these ideals.^17,18^ Whether through their own discretion, or because of resource limitations, many respondents attempted to deliver care in the confines of carceral settings., and were particularly narrow in their decisions to offer patients care in outside hospitals (where community standards would more likely be respected) than inside jail or prison infirmaries.

Without universal principles, ethical deliberations often became ad hoc and personal. Allocation of limited resources in life-or-death situations poses thorny ethical dilemmas.^8^ Participants described trade-offs, the need to develop new systems, and intense deliberations and stressful problem-solving in how they approached decisions related to movement. This included extended lockdowns or public health measures such as quarantine and isolation that restricted normal liberty and programming. The reported stressfulness of these decisions reveals not only the increased workload but also moral distress—the sense that not all valid claims to limited resources could be met and that therefore some degree of suffering and even loss of life might be linked to decisions made by staff.^9^

Despite high-stakes decisions confronting them, officials rarely invoked medical ethical principles (eg, maximizing overall utility, priority for the worst off) when describing their decision-making processes. In another paper, we noted this same concealment in how these officials dealt with liberty restrictions such as extended quarantines.^19^ Instead of talking directly about morally fraught trade-offs, participants preferred to frame their decisions as technical puzzles to be resolved. It may be that using ethical language, and explicitly weighing ethical principles, was not at the forefront of their minds, but rather operations and logistics were their primary concern. Another possibility is that individuals, faced with life-or-death decisions in a socially marginalized population, may seek to de-personalize the stakes, in essence to “sanitize” the moral complexity of the situation. From withholding vaccines for incarcerated populations to not enforcing vaccine mandates among staff, the politicization of the pandemic also appeared to influence decision-making. In the context of public health emergencies, there remains an important role for ethical analysis even when, often, there are no bright-line “wrong” or “right” decisions.

The literature on leadership in carceral facilities emphasizes the importance of strong and visionary leadership that leads by example and focuses on developing processes that work.^20,21^ The full-time presence of such creative leadership willing to negotiate the needs of stakeholders cannot be overestimated. While our study made no attempt to evaluate the quality of leadership, it was clear that many leaders marshaled creative solutions (eg, in use of space and medical equipment) in the face of unprecedented circumstances and took actions that exceeded the purview of their normal job duties. At some facilities, leaders engaged in active surveillance and preventive measures in conjunction with custody and health care staff. Another COVID-era study on how to improve pandemic preparedness in jails and prisons accentuated the need to include incarcerated people as key stakeholders in decision-making.^22^ While our study reveals creativity in making flexible adjustments, there were structural challenges in how rapidly resources could be adapted or deployed. Best practices also dictate that carceral facilities have ongoing negotiated relationships, contracts, or memoranda of understanding with local hospitals and specialty care centers to ensure access for their patients.^23^ Not all facilities seemed to have such agreements in place, however. Legislation or regulations mandating such relationships could prevent difficulties should detention centers face another public health emergency.

Additionally, in contrast to best practices in some hospitals that assembled multi-stakeholder groups (including representatives from the community and bioethicists), leadership fell upon medical and security leaders in prisons and jails.^24^ In other words, there was no counterweight to the biases of these groups. There appears to have been no external accountability through systematic external oversight by bodies that could have been created to promote ethical decision-making processes and the prevention of the operational problems identified in this paper. Such external bodies could have been created on the federal, state, and local levels and could have been composed of experts in the areas of infectious diseases, public health, carceral health, and bioethics. Interviews with incarcerated persons could be conducted by these oversight and advisory bodies to inform decision-making and increase transparency. In the interim, a set of general guidelines for public health emergencies in carceral settings could be created that focuses not only on clinical matters but on the day-to-day decisions that implicate the problems identified by the respondents in this study. Such guidelines could never be a one-size-fits-all solution nor substitute for facility-specific planning that included guidance on ethical issues, but could guide stakeholders as they navigate ethically thorny territory.

Several study limitations should be noted. First, our small sample size is not representative of all health care or custody officials, nor does it allow for adequate comparisons across different types of facilities. Second, the facilities come from a convenience sample and we cannot make any conclusions about generalizability. Third, the sample had more representation from health vs security or administrative leaders, which may offer an incomplete picture of how decisions were made (especially as security and administration can often override medical decisions). Fourth, interviews generally focused on only the first year of the pandemic response and therefore did not address how facilities have adapted as the COVID-19 variants continued to evolve and became more contagious and as new antiviral treatments were introduced. And finally, although interviews were designed to promote candid, honest conversations, interviewees may have experienced social desirability bias in how they described the measures taken and how they portrayed their own actions and emotions. In those interviews that were conducted as groups, we cannot determine whether peer effects or implicit social pressure could have swayed how individuals responded.

## Conclusion

The difficult, yet predictable, scale of COVID-19 outbreaks in jails and prisons highlights the importance of developing emergency-preparedness plans for a future pandemic, and ideally promoting more coordination across disparate carceral facilities. Planning based on the COVID-19 experience is critical. This includes focusing on how leaders weigh complex tradeoffs and communicate about the urgency of meeting the needs of incarcerated individuals. There is also a need to explicitly advocate for incarcerated people in planning for future pandemic events. Systematically drawing together lessons learned and formalizing decision-making around limited resources in planning documents is a step that carceral systems should be pursuing in anticipation of future pandemic events. Such planning now may promote more equitable decisions when confronted with a future pandemic event.

## Supplementary Material

qxae015_Supplementary_Data

## Appendix

**Appendix A.** Participant characteristics.

	No. of participants
Sex	
Male	13
Female	19
Role	
Medical	25
Custody	7
Place of employment	
Jail	19
State prison	5
Private vendor^a^	5
Juvenile detention	2
Immigration detention	1
Region of decision-making	
West	14
Midwest	2
South	7
Northeast	8
Nationwide	1

*n* = 32.

^a^“Private vendors” were individuals employed by medical vendors who worked across multiple facilities or in national headquarters.

**Appendix B.** Representative quotes.

Resource	Quote no.	Quote
Hospitalization	1	Respondent: “We had a handful of deaths. Those were tough to deal with. [It was] unclear as to who needed to be hospitalized and who didn’t. So that was difficult. Some people that were sent out and came back ended up going back to the hospital and dying or some died in our facilities.”—PID 111 (Medical Director)
Hospitalization	2	Respondent: “A lot of my time was spent negotiating with [community hospital] about accepting patients. Instead of treating patients, I was negotiating whether they could accept. So you’re kind of using me as a clinical administrator, rather than a clinician. It’s a subtle distinction. But, if I’ve got an hour, and I could see three patients during that time—that’s a better use of my time than me calling up another facility and saying, ‘I’ve got these six patients.’”—PID 109 (Doctor)
Masks	3	Respondent: “It was a really hard time getting everyone masks, getting people to wear masks. The COs (correctional officers) said that they were going to strike unless they got N95s, but we couldn’t get them N95s. So, there was there was the cloth, the surgical, the KN95, and the N95. And so, writing—I wrote a lot of memorandums that if you’re interacting with someone who has COVID, we want you to wear an N95. Otherwise, you know, a KN95 in the jail is okay, and permitted. It felt squabbling. It was so much time squabbling with unions.”—PID 101 (Doctor)
Masks	4	Respondent: “I think it really helped to be able to wear whatever mask you wanted. You know because, first of all, they (N95s) weren’t here and we were just doing the best we could to get what we could initially. But I think that made a big difference [with compliance] to allow that.”—PID 102 (Deputy Warden)
Physical space	5	Respondent: “In isolating inmates, you know, you can’t put certain inmates with other inmates. It was literally an ongoing, okay, can we move this high-custody inmate who’s only on day two of his quarantine in with this other inmate. How do you manage this?”—PID 117 (Warden)
Physical space	6	Respondent: “I know patients of mine died because, I don’t want to say because they were decarcerated, but they were rapidly pushed out without opioid use disorder treatment, not through the jail’s fault necessarily, but basically Fridays in March or April, they would come around and they would release tons of people. And then we couldn’t get their medication, their healthcare activated. And so, they would go out without the buprenorphine or the methadone that they needed. And then they would overdose and die.”—PID 101 (Doctor)
Vaccination	7	Respondent: “Phase 1 in [state] was clearly healthcare workers. So, when that happened for the jails, they were allowed to do health care workers. In some of the jails, if a CO was working in a health area, he could be considered a healthcare worker. For the employees that were not already vaccinated as a healthcare worker, this is where the first line thing comes in. There are some corrections officers that are also police officers, or fire marshals, etc. So, if you didn’t qualify in that first one, then they rolled out at the same time to the COs and the people who are incarcerated. That was the goal.”—PID 101 (Doctor)
Vaccination	8	Respondent: “I think the biggest thing to come out of [the pandemic] should be that if your incarcerated population is there and they end up having outbreaks, it’s not because of them. It’s because either you’re not testing them or it’s coming from the sheriffs and the guards because they’re the ones that go back and forth. Then, if you don’t end up dealing with that and testing them and masking them, the general public will be impacted because those infections are going to go back into the community. If you don’t treat the congregate population, whether they’re in a group home, juvenile jail, or jail, the whole community is going to suffer.”—PID 116 (Medical Director)
Vaccination	9	Respondent: “We decided early on that we weren’t going to vaccinate anybody outside of what the general public’s level was. So, when it first came out and, you know, you had to be a certain age with a pre-existing to get the vaccine. We had maybe five elderly inmates with a preexisting condition. So, those were the only five we offered it to. Mainly because, you know, we don’t need the community screaming at us for giving advantages to people who are incarcerated because they wouldn’t view it as fair.”—PID 107 (Chief Deputy)
COVID testing	10	Respondent: “The other determination that we made early on was we were going to test everybody in the vehicle sally [a double-gated area through which incoming and exiting vehicles travel through] prior to coming in. So that wasn’t just challenging for us. It’s also challenging for all law enforcement bringing people in because now we were holding them out in the vehicle sally until a nurse could get out there and rapid test the individual. So it slowed down their booking time, which means that they weren’t getting back out on the street. We had to navigate our way through that and say, no—we really need to do this.”—PID 105 (Public Health Nurse)
Staffing	11	Interviewer: “Are there any specific examples you can provide of how the security staff resistant to your recommendations and guidelines impacted your COVID response in a negative way in the beginning?” Respondent: “So, things like custody overrides, housing people in a gym. We set up tents and housed people in it. These were all directives coming from medical to custody, which were huge workloads but also, from their viewpoint, absolutely crazy. I had people living in libraries and all sorts of places around the facility in nontraditional housing units that they then had to place their officers to man those units and create more stations and overtime. Simultaneously, we were mapping their staff out.”—PID 127 (Medical Director)
Staffing	12	Respondent: “Anything that was felt to be routine was taken off the schedule. So the schedules were now just seeing the COVID-positives with COVID quarantine and the more urgent cases. All the routine stuff really got lagged behind for several months. And that certainly caused a problem. Dental care was also stopped. Optometry was stopped. So that was difficult. And, also, you know, unless it was really an urgent situation, it was very difficult to get any consultant to see the patients at this time. That caused many backlogs to develop in people getting seen both at the clinic level and, certainly, at the and at the consultant level. So that was a big challenge. And, now, we’re seeing a lot of people going out for consultants and we’re kind of catching up from what that was at that time.”—PID 111 (Medical Director)
